# Population Incidence and Mortality of Sepsis in an Urban African Setting, 2013–2016

**DOI:** 10.1093/cid/ciz1119

**Published:** 2019-11-14

**Authors:** Joseph M Lewis, Michael Abouyannis, Grace Katha, Mulinda Nyirenda, Grace Chatsika, Nicholas A Feasey, Jamie Rylance

**Affiliations:** 1 Malawi Liverpool Wellcome Clinical Research Programme, Blantyre, Malawi; 2 Liverpool School of Tropical Medicine, Liverpool, United Kingdom; 3 University of Liverpool, Liverpool, United Kingdom; 4 Adult Emergency and Trauma Center, Queen Elizabeth Central Hospital, Blantyre, Malawi; 5 University of Malawi, College of Medicine, Blantyre, Malawi

**Keywords:** sepsis, epidemiology, Africa south of the Sahara, low-resource setting

## Abstract

**Background:**

Sepsis is an important cause of mortality globally, although population incidence estimates from low-income settings, including sub-Saharan Africa, are absent. We aimed to estimate sepsis incidence burden using routinely available data from a large urban hospital in Malawi.

**Methods:**

We linked routine-care databases at Queen Elizabeth Central Hospital, Blantyre, Malawi, to provide admission and discharge data for 217 149 adults from 2013–2016. Using a definition of sepsis based on systemic inflammatory response syndrome criteria and Blantyre census population data, we calculated population incidence estimates of sepsis and severe sepsis and used negative binomial regression to assess for trends over time. Missing data were multiply imputed with chained equations.

**Results:**

We estimate that the incidence rate of emergency department–attending sepsis and severe sepsis in adults was 1772 per 100 000 person-years (95% confidence interval [CI], 1754–1789) and 303 per 100 000 person-years (95% CI, 295–310), respectively, between 2013 and 2016, with a year-on-year decrease in incidence. In-hospital mortality for patients admitted to the hospital with sepsis and severe sepsis was 23.7% (95% CI, 22.7–24.7%) and 28.1% (95% CI, 26.1 – 30.0%), respectively, with no clear change over time.

**Conclusions:**

Sepsis incidence is higher in Blantyre, Malawi, than in high-income settings, from where the majority of sepsis incidence data are derived. Worldwide sepsis burden is likely to be underestimated, and data from low-income countries are needed to inform the public health response.

## Teaser

Sepsis epidemiology in sub-Saharan Africa (sSA) is poorly described. Using large databases from a Malawian teaching hospital, we present some of the first estimates of sepsis population incidence from sSA and find it to be high compared with high-income settings.

Sepsis, recently redefined as a syndrome of life-threatening organ dysfunction triggered by infection [1], is a significant public health problem, with an estimated 31.5 million cases worldwide and 5.3 million deaths [2]. However, these figures are at best rough approximations: population incidence data for low-income settings, including sub-Saharan Africa (sSA), are absent, and there are reasons to suspect that the burden of sepsis may be higher in low-income settings. In Africa, febrile illness is common: malaria, for example, had an estimated incidence of 219.4 cases per 1000 person-years at risk in 2017 [3]. Although sepsis is not included in global burden of disease estimates, infections such as pneumonia [4], meningitis [5], and typhoid fever [6] have persistently higher incidence rates in low- and middle-income countries than in high-income countries. In addition, the few available cohort studies suggest that the case fatality rates of sepsis in sSA are higher than in high-income settings [7, 8].

By using a routinely collected dataset of over 200 000 emergency department (ED) attendances from a single urban African center, we aimed to estimate the overall and age-stratified population incidence of sepsis in Blantyre, Malawi, to describe changes over a 4-year period and to estimate inpatient sepsis case fatality rate.

## METHODS

### Setting and Data Sources

Malawi is a low-income country in Southeast Africa. It has a high burden of human immunodeficiency virus (HIV) and tuberculosis (TB) as defined by the World Health Organization, with an adult (age 15–49 years) HIV prevalence of 9.6% in 2016 [9]. Healthcare is free to all at the point of delivery (including TB and malaria treatment) and free antiretroviral treatment (ART) has been available since 2004. Malawi has a hot, wet season from November to March and well-recognized seasonal disease patterns. Malaria is endemic but with peak incidence during the rainy season from November to May [10], with peaks of invasive *Salmonella* infection during and after the rainy season [11] and invasive pneumococcal disease in the dry season [12].

Queen Elizabeth Central Hospital (QECH), Blantyre, Malawi, has 1000 beds and is the only government hospital providing free inpatient medical care to the city of Blantyre (population 800 024 at the 2018 census). The majority of adult inpatients have HIV; previous studies have found 75–90% of patients with sepsis, fever, meningitis, or pneumonia to have HIV [13–16]. Adults are admitted via a dedicated ED. Despite its tertiary facility status, most attendees are either primary presentation or referrals from primary health care clinics; interhospital transfers are not common. In 2011, triage was introduced, and an electronic patient registration system was established in September 2012, recording basic patient demographics and vital signs before directing staff to the most appropriate triage category. Additionally, an independent limited electronic patient record has been used to record vital status and diagnostic information at patient discharge for those admitted to the wards (but not those directly discharged from the ED) [17].

We used anonymized data from both electronic systems, and matched patients on unique system identifiers and dates of attendance for a 4-year period (2013–2016). Human immunodeficiency virus status and details of any investigations (blood tests, radiology, or microbiology) were not available. We included all ED-attending patients aged 14 years or over and all patients in electronic discharge records who could be linked to an admission record.

For Malawi population data we retrieved the Malawian census data for Blantyre city for 2008 and 2018 from the National Statistical Office of Malawi [18]. To extrapolate between these 2 time points we fitted a log-transformed time curve using a linear model. Age distribution within Blantyre city was not available for 2018; we extrapolated age-specific denominators from national data on pooled urban populations.

### Statistical Analysis

We defined sepsis using a modified Sepsis-2 definition [19] as fever (>38°C) or hypothermia (<36°C) plus one of tachycardia (heart rate >90 beats/minute) or tachypnea (respiratory rate >22 breaths/minute). We defined severe sepsis as sepsis plus one of shock (systolic blood pressure <90 mmHg), hypoxia (oxygen saturation [SpO_2_] <90%), or reduced consciousness level (below A on the alert, voice-responsive, pain-responsive, unresponsive scale). Due to the fact that our data were not missing completely at random (Supplementary Figures 2 and 3), we performed multiple imputation, a procedure that reduces bias at the cost of introducing imprecision. We used chained equations in the *mice* package [20] in R (R Foundation for Statistical Computing); systolic and diastolic blood pressure, heart rate, oxygen saturation, respiratory rate, consciousness level, age, and sex were used to predict all missing variables, generating 5 imputed datasets.

To calculate population incidence rates of ED-attending sepsis and severe sepsis we first generated pooled absolute numbers and (assuming a Poisson distribution) variances of cases of sepsis and severe sepsis from the 5 imputed datasets using Rubin’s rules [21]. To calculate population incidence, we assumed that the calculated population of Blantyre city was at risk and used the pooled variance to construct confidence intervals (CIs). Age- and period-specific incidences were similarly calculated using the estimated denominators as above. To produce estimates of inpatient sepsis and severe sepsis mortality we pooled mortality estimates and variances from the 5 imputed datasets. In order to assess for time trends in sepsis incidence, we performed negative binomial regression with the a priori covariates of year as a continuous variable and rainy season (defined as November–March) as a binary variable. The output of these models were expressed as adjusted incident rate ratios (aIRRs) per year increase or for rainy season compared with dry season.

In view of the high proportion of missing data, we performed a number of sensitivity analysis. We produced maximum and minimum population incidence estimates by assuming that all missing values were clinically normal or clinically abnormal, respectively, and we produced complete-case incidence estimates by extrapolating from those records that had no missing data by assuming that the proportion of participants with sepsis or severe sepsis was the same in the population with missing data as those with complete data. We also explored the effect of differing sepsis definition, defining sepsis as a quick Sequential Organ Failure Assessment [22] score of 2 or more, a Universal Vital Assessment score [23] of 2–4 and 4 or more, and a Modified Early Warning Score [24] of 5 or greater and calculating incidence using imputed data as above.

All analyses were carried out on R version 3.5.1.

### Ethical Approval

This study used only anonymized, routinely collected data. It was approved by the Malawi College of Medicine Research Ethics Committee (P.01/18/2331)

## RESULTS

From 1 January 2013 to 31 December 2016 there were 217 149 unique adult attendances to the ED recorded in the triage system. There were varying proportions of missing data for these attendances. The majority of variables had less than 5% missing data, but 3 variables had a significant proportion of missing data (Supplementary Figure 1), as follows: respiratory rate (82% missing), oxygen saturation (65% missing), and consciousness level (93% missing). Demographic characteristics of the patients in complete-case analysis calculated from the unimputed dataset are shown in Table 1.

**Table 1.** Characteristics of Included Patients from Complete-Case Analysis

	Nonsepsis		Sepsis		Severe Sepsis	
	Value	Not Missing Data, n	Value	Not Missing Data, n	Value	Not Missing Data, n
Total n	…	187 644	…	29 505	…	3700
Age, years	33.2 (25.1–45.4)	187 644	33.6 (25.1–45.5)	29 505	35.3 (27.3–46.4)	3700
Male, %	48	187 644	45	29 505	49	3700
Temperature, °C	36.4 (36–36.9)	179 780	35.6 (35.3–38.4)	29 505	35.6 (35.3–38.4)	3700
HR, beats/minute	88 (77–103)	182 507	108 (98–123)	29 367	115 (102–130)	3684
RR, breaths/minute	20 (18–22)	39 718	22 (19–25)	7822	22 (19–25)	1134
SBP, mmHg	121 (109–136)	180 731	115 (101–131)	28 313	84 (77–89)	3567
DBP, mmHg	78 (69–87)	180 742	75 (65–86)	28 323	57 (49–65)	3566
SpO_2_, %	98 (96–99)	64 841	97 (95–99)	11 273	93 (85–97)	2001
Alert, %	97	12 845	97	1415	80	218

Data are presented as median (IQR) unless otherwise indicated.

Abbreviations: DBP, diastolic blood pressure; HR, heart rate; IQR, interquartile range; RR, respiratory rate; SBP, systolic blood pressure; SpO_2_, oxygen saturation.

### Population Incidence

**Figure 1.**
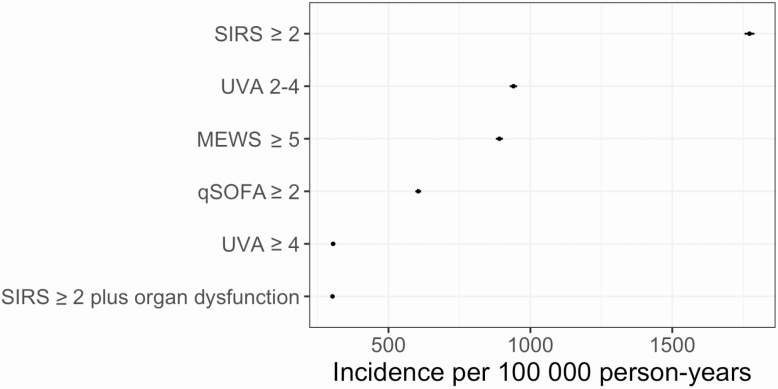
Sensitivity analysis showing estimated sepsis population incidence with varying definitions of sepsis. Abbreviations: MEWS, modified Early Warning Score; qSOFA, quick sequential organ failure assessment; SIRS, systemic inflammatory response syndrome; UVA, Universal Vital Assessment.

Estimates of adult population incidence rates of sepsis and severe sepsis were 1772 per 100 000 population (95% CI, 1754–1789) and 303 per 100 000 population (95% CI, 295–310), respectively, following multiple imputation of missing data. Sensitivity analysis assuming that missing data were clinically normal or abnormal gave minimum and maximum estimates (Table 2); differing sepsis definitions gave varying estimates of incidence (Figure 1, Supplementary Table 1).

**Table 2.** Sensitivity Analyses Showing Minimum and Maximum Bounds of Sepsis and Severe Sepsis Incidence Assuming All Missing Values Are Clinically Normal or Abnormal, Respectively

Estimate	Incidence/100 000 Population
Sepsis (minimum)	1378
Sepsis (complete case)	1711
Sepsis (maximum)	2741
Severe sepsis (minimum)	173
Severe sepsis (complete case)	239
Severe sepsis (maximum)	2666

**Figure 2.**
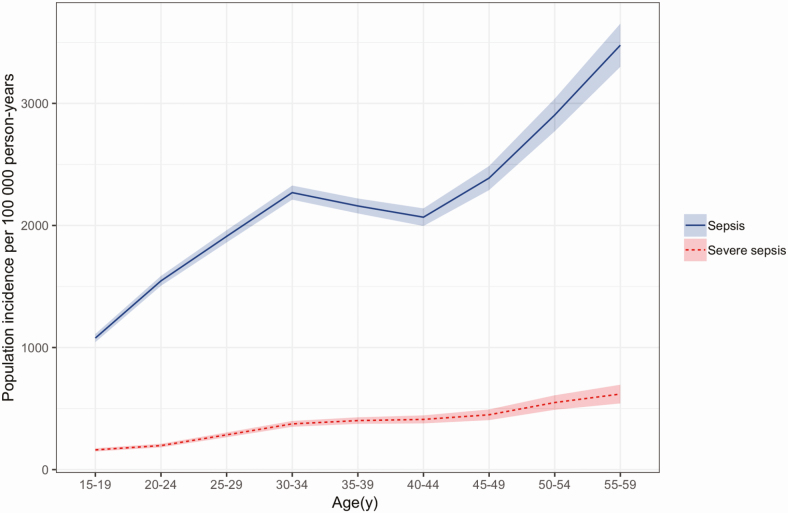
Age-stratified population incidence rate of adult sepsis and severe sepsis for Blantyre, Malawi, 2013–2016, per 100 000 person-years at risk.

Age-stratified population incidence rate showed an increasing incidence with increasing age (Figure 2). The lowest estimates were observed in the 15- to 19-year group (sepsis, 1077; 95% CI, 1044–1111; severe sepsis, 162; 95% CI, 148–175 cases per 100 000 person-years) and highest in those aged 80 or older (sepsis, 9726; 95% CI, 9075–10 377; severe sepsis, 2528; 95% CI, 2183–2872 cases per 100 000 person-years).

**Figure 3.**
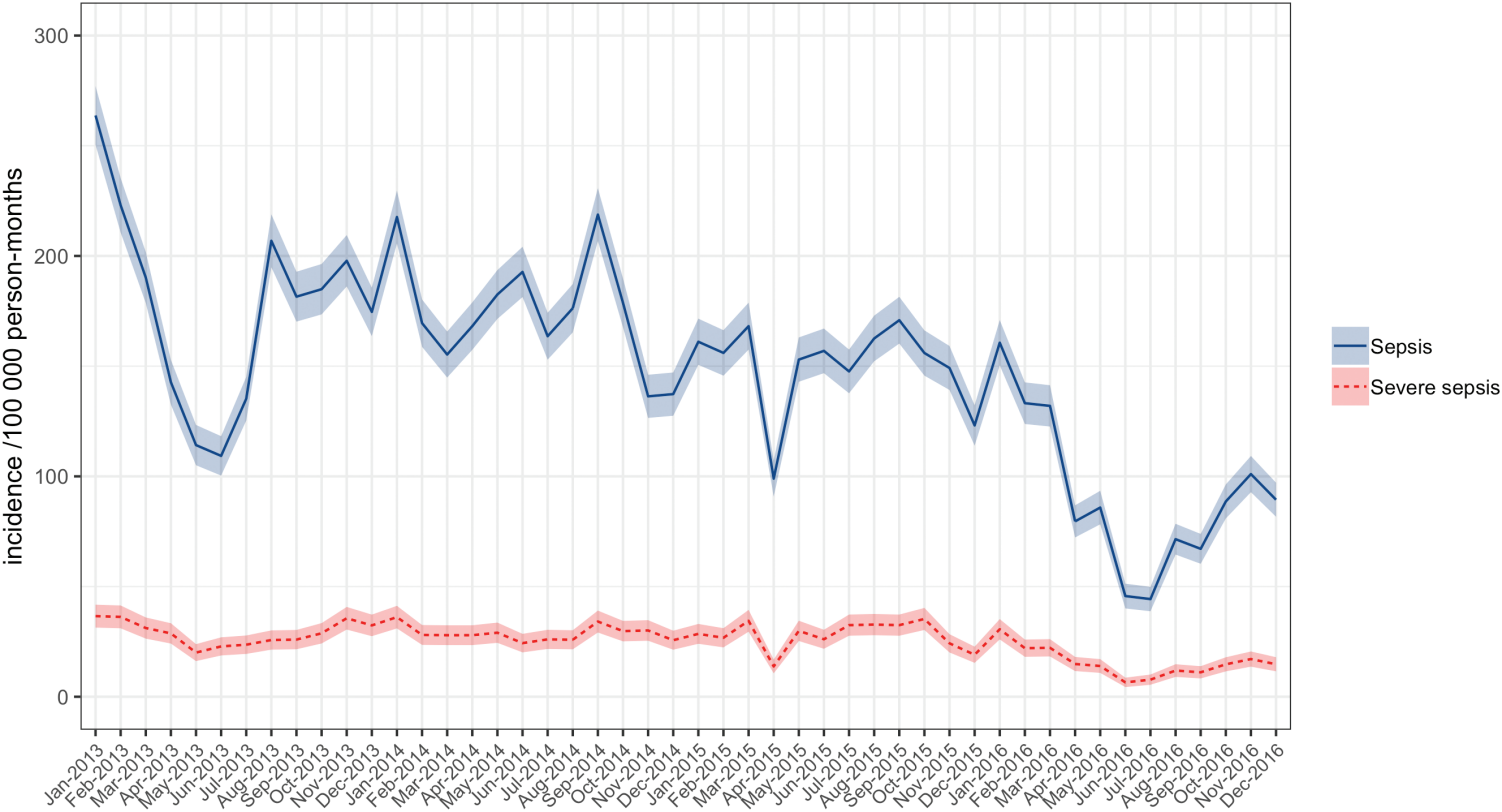
Population incidence of adult sepsis (blue solid line) and severe sepsis (dotted red line) per 100 000 person-months at risk as a function of time.

The population incidence of sepsis almost halved over the study period (Figure 3), from 2124 (95% CI, 2085–2163) sepsis and 348 (95% CI, 332–364) severe sepsis cases per 100 000 person-years in 2013 to 1099 (95% CI, 1072–1126) sepsis and 187 (95% CI, 176–199) severe sepsis cases per 100 000 person-years in 2016, with an aIRR of .81 (95% CI, .78–.84; *P* < .001) per year for sepsis and an aIRR of .84 (95% CI, .82–.87; *P* < .001) per year for severe sepsis in multivariable negative binomial regression. There was, in addition, a suggestion of a seasonal pattern, with the incidence higher in the wet season (November–March); although this pattern appeared to be inconsistent from year to year (Figure 2), multivariable negative binomial regression models found an independent association between rainy season versus dry season for sepsis (aIRR, 1.11; 95% CI, 1.04–1.19; *P* = .002) and severe sepsis (aIRR, 1.13; 95% CI, 1.06–1.21; *P* < .001).

### Inpatient Outcome

**Figure 4.**
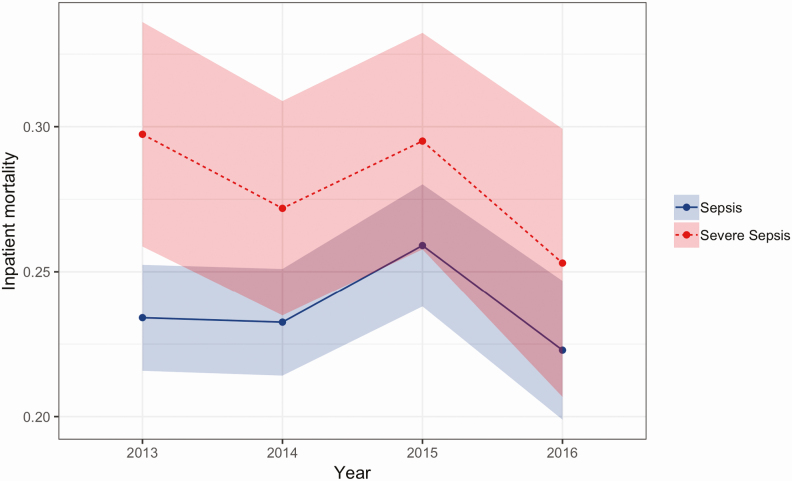
Sepsis (blue solid line) and severe sepsis (red dotted line) inpatient mortality as a function of time. Shaded areas are 95% confidence intervals.

**Figure 5.**
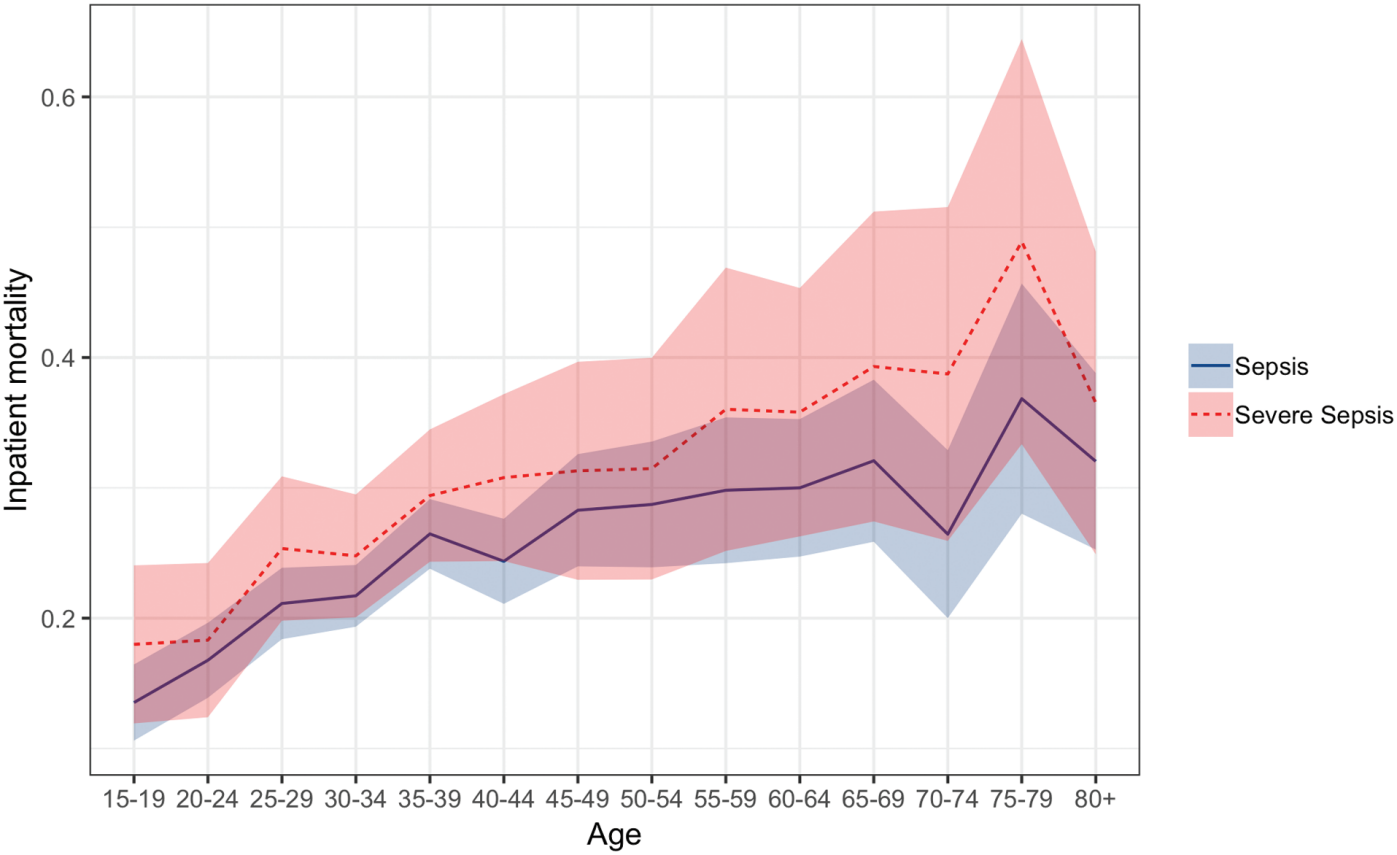
Sepsis (blue solid line) and severe sepsis (red dotted line) age-stratified inpatient mortality. Shaded areas are 95% confidence intervals.

For the period 2013–2016 outcome data were available for 23 787 adult inpatients. Of these, 21 136 (89%) could be matched to a unique record in the ED that provided admission vital signs. Of these matched records, 4810 patients died. Pooled inpatient sepsis mortality following imputation of missing data for the entire period was 23.7% (95% CI, 22.7–24.7%), and severe sepsis mortality was 28.1% (95% CI, 26.1–30.0%). Despite the reduction in population incidence of sepsis and severe sepsis, there was no apparent mortality trend for sepsis or severe sepsis by year, with overlapping CIs for mortality rate estimates for all years 2013–2016 (Figure 4). Mortality increased with age for both sepsis and severe sepsis, increasing with age up to approximately age 35 and then more slowly with age thereafter (Figure 5).

## DISCUSSION

We estimate the headline population incidence of ED-attending sepsis and severe sepsis to be 1772 per 100 000 population (95% CI, 1754–1789) and 303 per 100 000 population (95% CI, 295–310) in Blantyre, Malawi, from 2013 to 2016. In addition, the population presenting with sepsis is young, with a median age of 34 years (sepsis) or 35 years (severe sepsis).

Estimates of sepsis mortality from high-income settings vary depending on the definitions and coding strategies, making direct comparisons difficult. A recent meta-analysis of 27 studies [2] from 7 high-income countries found an incidence rate of 437 (95% CI, 334–571) for sepsis and 270 (95% CI, 176–412) for severe sepsis cases per 100 000 person-years. Hospital mortality was 17% for sepsis and 26% for severe sepsis, but these estimates were largely derived from discharge databases. These may not be directly comparable because diagnoses are often based on classification systems such as the *International Classification of Diseases*, *10th Revision*, rather than admission physiology and patients are largely admitted to hospital (rather than ED attendees, as here). Two studies calculated the population incidence of ED-treated sepsis/severe sepsis, which may be more comparable, estimating a sepsis incidence of 731 cases per 100 000 person-years in Denmark [25] and a severe sepsis incidence of 140 and 265 cases per 100 000 person-years in the United States [26] and Denmark [25], respectively. The participants presenting with sepsis in high-income settings in the systematic review had a median age ranging from 47 to 73 years, which is considerably higher than what we report from Malawi. One study [27] estimated intensive therapy unit (ITU)-treated sepsis incidence in Brazil—a middle-income country—and found it to be 290 (95% CI, 238–351) adult cases per 100 000 population per year, comparable to our estimate of ED-attending severe sepsis.

It is therefore probable—especially given the paucity of variables that were available in our study to define sepsis and severe sepsis—that the incidence of sepsis in Blantyre, Malawi, is higher than in high-income settings. In addition, sepsis is occurring in a younger population, and despite this, mortality remains high. The reasons for this are not apparent from our data. Human immunodeficiency virus may play a role, and other prevalent pathogens such as TB or malaria may also be implicated. Human immunodeficiency virus is most prevalent in young adults and hence may drive sepsis presentation at a younger age. It could also contribute to the blunting of the usual inpatient relationship between age and mortality that we describe; inpatient sepsis mortality for the 35- to 40-year-old age group was not dramatically different from the age group 80 years or older in this dataset: for example, 26.4% (95% CI, 23.8–29.1%) versus 32.0% (95% CI, 25.3–38.8%). In high-income settings, mortality in the elderly might be expected to be significantly higher than in young adults: in 1 large study in the United States, for example, sepsis mortality was 18.0% in those aged 80 years or older versus 8.6% in the 20- to-39-year age group [28].

We saw a clear trend in decline in sepsis incidence over time, with a superimposed seasonal pattern and no clear change in case fatality rate. Our data provide no insight into the drivers of this phenomenon, although there are several possible explanations. First, this could represent a true change in sepsis incidence. Significant progress has been made in Malawi with both control of HIV (through antiretroviral provision) and malaria, both of which could contribute to a decrease in sepsis incidence. Indeed, life expectancy at birth in Malawi has increased from 42 years in 2000 to 64 in 2017 [29], which is consistent with the significant reduction in incidence in sepsis that we have seen in this study. Alternatively, changes in referral patterns, or coding or data entry practices, might introduce artefactual error. We are aware of at least 1 quality-improvement program at one of the health centers that refer to QECH that occurred during the study period, for example. Despite the apparent reduction in incidence, inpatient mortality remained unchanged, highlighting the fact that sepsis, once established, carries a high mortality and the need for improved quality of care from recognition to management in the Malawian setting. The seasonal pattern we observed is likely related to well-recognized patterns in the causes of febrile illness in Malawi, with a higher incidence of invasive *Salmonella* —an important pathogen in this setting [30]—in the rainy season, for example [11].

There are significant limitations to our study. Most notably, there were significant proportions of missing data. We have used imputation to reduce bias compared with simpler imputation schemes, such as assuming clinically normal data for missing values, but this is at the expense of imprecision. We used a modified Sepsis-2 definition of sepsis [19] using the systemic inflammatory response syndrome (SIRS) criteria, despite the publication of newer (Sepsis-3) definitions. The components of the new definition (the sequential organ dysfunction assessment or SOFA score [1]) were not available in our databases, and indeed are rarely available in routine clinical care in our hospital. Furthermore, Sepsis-2–based incidence estimates provide some “backward compatibility” with previous estimates from high-income settings for comparison. True Sepsis-2 sepsis definitions require a suspicion or confirmation of infection plus SIRS components; these were lacking and we used the presence of fever or hypothermia as a proxy. This might overestimate sepsis incidence, although in clinical practice in Malawi the presence of fever constitutes sufficient evidence to suspect infection. In defining severe sepsis, we were again limited by the data available to us and used a relatively narrow definition of shock, low oxygen saturations, or low consciousness level; our estimates of severe sepsis are therefore very likely to be an underestimate. Although we produced population incidence estimates and mortality for sepsis and severe sepsis, these 2 populations are subtly different: incidence calculations are for ED-attending sepsis/severe sepsis, whereas mortality is for patients who have been admitted to the wards. The mortality for ED attendees is unknown but would be expected to be lower.

There were other limitations arising from our use of routinely captured data. The databases we used would not capture patients who died in the ED; this could result in an underestimate of sepsis mortality. However, and although our data cannot address this question, our experience of the ED at QECH suggests that death in the ED is uncommon. Many patients with sepsis will have been discharged from the ED (either with or against medical advice) and managed as outpatients. If they died at home they would not be captured in our sepsis mortality estimates; furthermore, in the Malawian setting, it is likely that some people develop sepsis at home and die without seeking healthcare. They, too, would not be captured either in our incidence or mortality estimates. Some participants recorded in the database with apparently unique patient IDs may, in fact, represent re-registration of the same individual. The databases we used are not linked to investigations performed, so HIV status of participants and etiology of sepsis are unknown.

In conclusion, we have used a large dataset from a single African center to calculate the first, to our knowledge, population incidence estimates for sepsis for a country in sSA. We found that sepsis is common, affecting a young population, and, despite this, carries a high mortality. Previous estimates of 31 million sepsis cases, 19.4 million severe sepsis cases, and 5.3 million deaths are extrapolated from high-income settings to low- and middle-income settings, where 83% of the world’s population live [2], and are very likely underestimates. A speculative extrapolation of our findings to all low- and middle-income countries suggests that the worldwide incidence could be as high as 111.4 million sepsis cases and 21.4 million severe sepsis cases. This very wide disparity in estimates highlights the problems with extrapolating incidence estimates across very different settings. It emphasizes the need for data to guide the public health response to sepsis in sSA and other low-income settings and to describe the basic epidemiology but also develop interventional strategies that can be deployed at scale in low-resource settings.

## Supplementary Data

Supplementary materials are available at *Clinical Infectious Diseases* online. Consisting of data provided by the authors to benefit the reader, the posted materials are not copyedited and are the sole responsibility of the authors, so questions or comments should be addressed to the corresponding author.

ciz1119_suppl_Supplementary_Material

## Notes

***Disclaimer.*** The views expressed are those of the authors and not necessarily those of the National Health Service (NHS), the National Institute for Health Research, or the Department of Health and Social Care.

***Financial support.*** This article was funded by the Department for International Development/Medical Research Council/Wellcome Joint Global Health Trials (grant number MR/P020577/1). Additional funding was received by the National Institute for Health Research using Official Development Assistance funding (the African Research Collaboration on Sepsis, 17/63/42). J. M. L. is supported by a Wellcome Trust clinical PhD fellowship (109105z/15/a). M. A. is supported by a Wellcome Trust clinical PhD fellowship (203919/Z/16/Z).

***Potential conflicts of interest.*** The authors: No reported conflicts of interest. All authors have submitted the ICMJE Form for Disclosure of Potential Conflicts of Interest.
