# Supplementary material for: Population Incidence and Mortality of Sepsis in an Urban African Setting, 2013–2016
Source: Clin Infect Dis. 2019 Nov 14;71(10):2547–52. doi: 10.1093/cid/ciz1119 (PMC7744994; doi:10.1093/cid/ciz1119)
Supplement: ciz1119_suppl_Supplementary_Material [file ciz1119_suppl_supplementary_material.docx]

**Supplementary material**


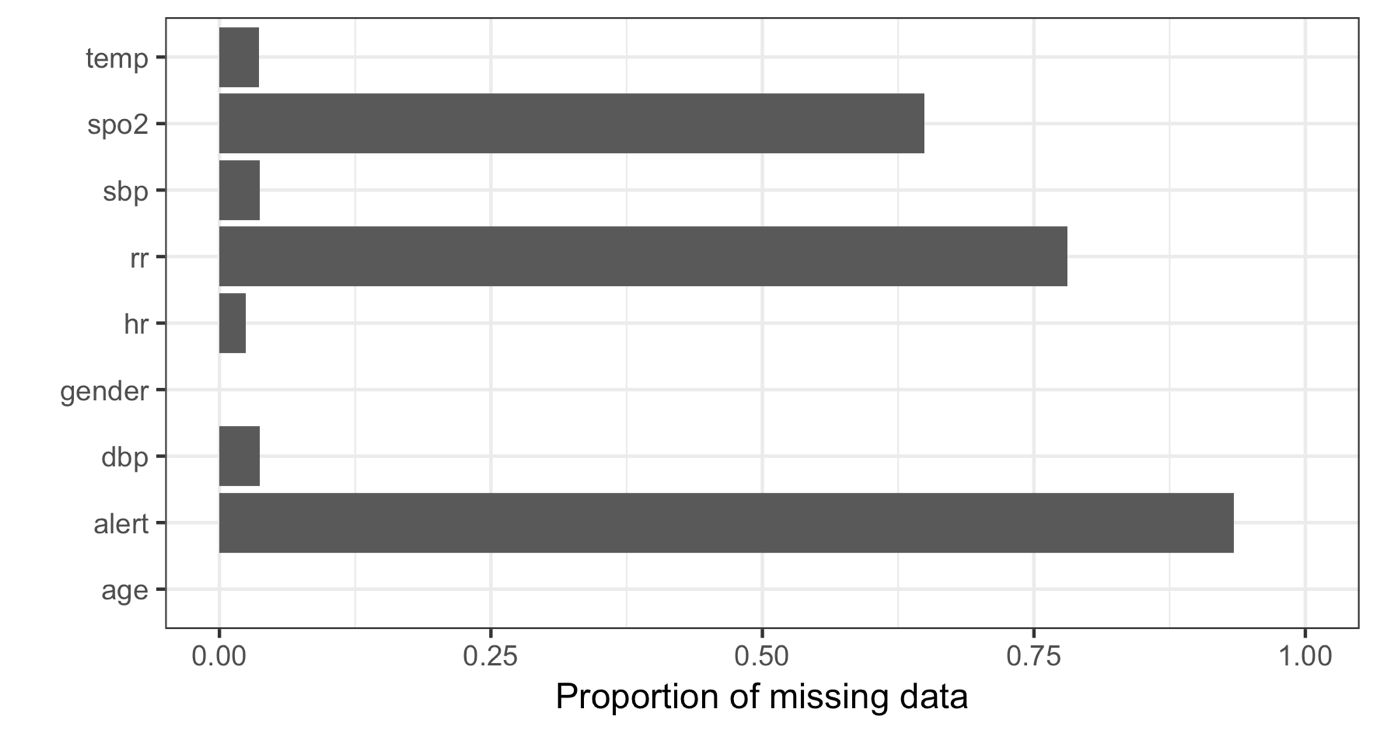


**Supplementary Figure 1:** missing data by variable.


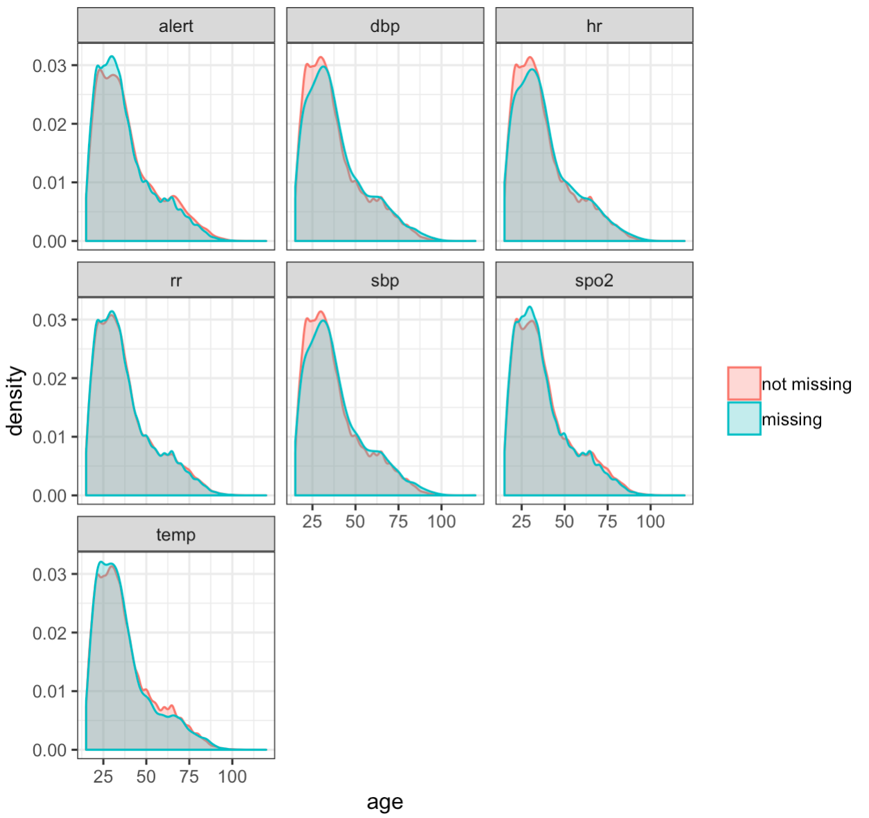


| Variable | p.value |
| --- | --- |
| alert | <0.001 |
| dbp | <0.001 |
| hr | 0.003 |
| rr | 0.015 |
| sbp | <0.001 |
| spo2 | <0.001 |
| temp | <0.001 |

**Supplementary Figure 2:** Age distribution of participants missing or not missing each of the 7 variables used in the analyis stratified by missing or not missing (top) and p values from a regression model predicting missingness in each of the 7 variables using age and gender as predictors (bottom); the p values test the hypothesis that the odds ratio of a missing value the given variable for a one year increase in the age is different to 1. This shows that the data is not missing completely at random and a complete-case analysis is likely to produce biased results


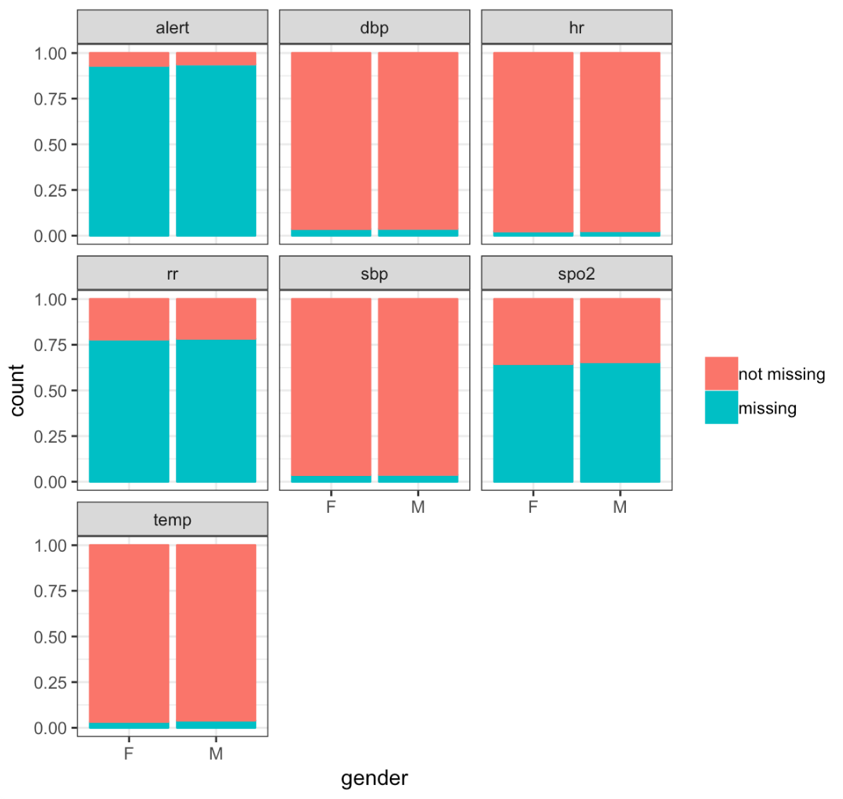


| Variable | p.value |
| --- | --- |
| alert | <0.001 |
| dbp | 0.572 |
| hr | 0.052 |
| rr | 0.008 |
| sbp | 0.549 |
| spo2 | <0.001 |
| temp | <0.001 |

**Supplementary Figure 3:** Proportion of missing values for each of the 7 variables used in the analysis stratified by gender (left) and p values from a regression model predicting missingness in each of the 7 variables using age and gender as predictors; the p values test the hypothesis that the odds ratio of a missing value in the variable for males compared to females is different to 1. This shows that the data is not missing completely at random and a complete-case analysis is likely to produce biased results

| Sepsis definition | Population incidence |
| --- | --- |
| SIRS >= 2 plus organ dysfunction | 303 (295-310) |
| UVA >= 4 | 305 (297-312) |
| qSOFA >= 2 | 605 (594-615) |
| MEWS >= 5 | 891 (878-903) |
| UVA 2-4 | 940 (927-953) |
| SIRS >= 2 | 1772 (1754-1789) |

**Supplementary Table 1:** Sensitivity analysis showing estimated sepsis population incidence with varying definitions of sepsis. SIRS = Systemic inflammatory response syndrome; UVA = Universal Vital Assessment; qSOFA = quick sequential organ failure assessment; MEWS – modified early warning score.
